# Stereotactic biopsies of brainstem lesions: which approach?

**DOI:** 10.1007/s00701-021-04733-2

**Published:** 2021-02-04

**Authors:** Amer Jaradat, Andreas Nowacki, Jens Fichtner, Janine-Ai Schlaeppi, Claudio Pollo

**Affiliations:** Department of Neurosurgery, Inselspital, University Hospital Bern, and University of Bern, Freiburgstrasse, 3010 Bern, Switzerland

**Keywords:** Brainstem, Stereotactic biopsy, Frame-guided, Stereotactic trajectory

## Abstract

**Background:**

Stereotactic biopsies for brainstem lesions are frequently performed to yield an accurate diagnosis and help guide subsequent management. In this study, we summarize our experience with different stereotactic approaches to brainstem lesions of different locations and discuss possible implications for safety and diagnostic yield.

**Methods:**

We retrospectively analyzed 23 adult patients who underwent a stereotactic biopsy for brainstem lesions between October 2011 and December 2019. Depending on the location supra- or infratentorial, trajectories were planned. We assessed the postoperative complications during the hospital stay as well as the diagnostic yield.

**Results:**

A supratentorial transfrontal approach was used in 16 (70%) cases, predominantly for lesions in the midbrain, upper pons, and medulla oblongata. An infratentorial, transcerebellar-transpeduncular approach was used in 7 (30%) cases mainly for lesions within the lower pons. All biopsies were confirmed to represent pathological tissue and a definitive diagnosis was achieved in 21 cases (91%). Three patients (13%) had transient weakness in the contralateral part of the body in the immediate postoperative period, which improved spontaneously. There was no permanent morbidity or mortality in this series of patients.

**Conclusion:**

Lesions of various locations within the brainstem can be successfully targeted via either a supratentorial transfrontal or an infratentorial transcerebellar transpeduncular approach. Our high diagnostic yield of over 90% and the low rate of complications underlines the diagnostic importance of this procedure in order to guide the medical management of these patients.

## Introduction

Brainstem lesions comprise 10–15% of all space occupying lesions of the brain in children and 2% in adult patients [13, 15]. Many diffuse brainstem lesions in the pediatric population do not warrant a biopsy due to the typical appearance on MR imaging suggesting a diffuse brainstem glioma with no relevant differential diagnoses [19]. In adults, however, pure radiological findings will often fail to make the correct diagnosis, due to the broader spectrum of underlying diseases including gliomas, tumors of other origin, infectious diseases, infarctions, vasculitis, demyelinating diseases, and gliosis [1, 6]. In fact, MRI-based diagnosis is reported to be erroneous in 10–20% of the cases [12]. Furthermore, the MRI accuracy to assess the tumor classification and grading was estimated to be correct in only 35% of low-grade gliomas and 27% of high-grade gliomas [17]. With growing importance of molecular markers for tumor classification and guidance of treatment, together with increasing safety and diagnostic yield of stereotactic biopsy of brainstem lesions, the justification of specimen-based diagnosis seems ever more important even in pediatrics population, as the availability of biopsy material will allow for molecular biology analysis including whole genome sequencing, thus potentially allowing for the development of future therapies [15, 18]. Stereotactic biopsies of brainstem lesions can be performed along different trajectories depending mainly on the location of the lesion. Both supratentorial and transcerebellar approaches have been described with similar outcomes [22]. In this study, we report our experience in 23 brainstem biopsies in an adult population, including the spectrum of diagnosis, the chosen trajectory, and the complications. Secondarily, we aim to propose surgical biopsy trajectories (i.e., supratentorial transfrontal or infratentorial transcerebellar, transpeduncular) according to the anatomical location of the lesion.

## Material and method

### Patient population

A consecutive series of twenty-three adult patients (16 males, 7 females) who underwent a stereotactic biopsy for a brainstem lesion at the University Hospital Bern between October 2011and December 2019 are included in this retrospective study. All adult patients who present with a brainstem lesions without clear radiological or previously known diagnosis and/or which were not amenable to surgical resection were selected for a stereotactic biopsy to confirm the diagnosis in order to direct multidisciplinary treatment plans .The patients’ mean age is 58 years (ranging 28 to 82 years) All cases of radiologically demonstrated lesions localized to brainstem were included in the study. In patients with larger lesions involving other regions of brain, in addition to brainstem, or multiple lesions, only the cases where the target of biopsy was brainstem were included. All cases with a target outside the brainstem were excluded. The mean duration of symptoms was 4.2 months. Symptoms consisted of cranial nerve deficits in 6, limb weakness in 5, gait disturbance in 3 patients, headache and signs of increase intracranial pressure in 4, sensory disturbances in 3, and 2 patients were not documented. Patient’s demographic, clinical presentation, chosen trajectory, histological diagnosis, and complications are summarized in Table 1. This study has been approved by the local ethics committee (KEK number 2020-00440).

**Table 1:** Patient’s demographic, clinical presentation, chosen trajectory, histological diagnosis, and complications

Patient	Age/gender	Localization	Presenting symptoms	Approach	Complications	Histopathological diagnosis
1	39/F	Diffuse pontomedullary	Double image	Transfrontal	None	Astrocytoma WHO grade II
2	76/F	Pons	hemiparesis	Transcerebellar	None	EBV-associated CNS lymphoma
3	56/M	Pontomesencephalone	Hemiparesis, gait instability	Transcerebellar	None	GBM, WHO grade IV
4	71/M	Midbrain tegmentum	headache	Transfrontal	None	GBM, WHO grade IV
5	78/M	Pontomedullary	Double image	Transcerebellar	None	Lymphoma
6	28/M	Pontomedullary	Paresthesia, dizziness	Transcerebellar	None	Anaplastic astrocytoma WHO grade III
7	75/M	Pontomesencephalone	Double image	Transfrontal	Right sided weakness	Anaplastic astrocytoma WHO grade III
8	58/M	Pontomesencephalone	Headache	Transfrontal	None	Anaplastic astrocytoma WHO grade III
9	50/M	Diffuse brainstem	Gait instability	Transfrontal	None	Anaplastic astrocytoma WHO grade III
10	70/M	Midbrain	Double image	Transfrontal	upper limb weakness	CNS lymphoma
11	63/M	Midbrain tegmentum	Hemiparesis	Transfrontal	None	GBM, WHO grade IV
12	82/F	Midbrain	Double image, gait instability	Transfrontal	None	B-cell lymphoma
13	73/M	Midbrain tectum	Headache	Transfrontal	upper limb weakness	GBM, WHO grade IV
14	66/F	Midbrain	Headache, double vision,	Transfrontal	None	Metastaric neuroendocrine carcinoma (colon CA)
15	67/M	Pontomedullary	Paresthesia	Transcerebellar	None	B cell lymphoma
16	31/F	Midbrain tegmentum	Dizziness	Transfrontal	None	Hypercellularity (Rosenthal fibers)
17	66/M	Midbrain	Not documented	Transfrontal	None	Anaplastic astrocytoma WHO grade III
18	55/M	Pontomedullary	Dysphagia	Transcerebellar	None	GBM WHO grade-IV
19	45/M	Medulla oblongata	Headache	Transcerebellar	None	Ependymoma WHO grade II
20	37/F	Pons	Headache, double image	Transfrontal	None	Pilocytic astrocytoma
21	40/M	Midbrain	Gait disturbances	Transfrontal	None	Nonspecific gliosis with microhemorrhage
22	60/M	Pontomesencephalone	Not documented	Transfrontal	None	GBM WHO grade IV
23	68/M	Pontomesencephalone	Paresthesia	Transfrontal	None	GBM WHO grade IV

### Pre-operative imaging

**Fig. 1:**
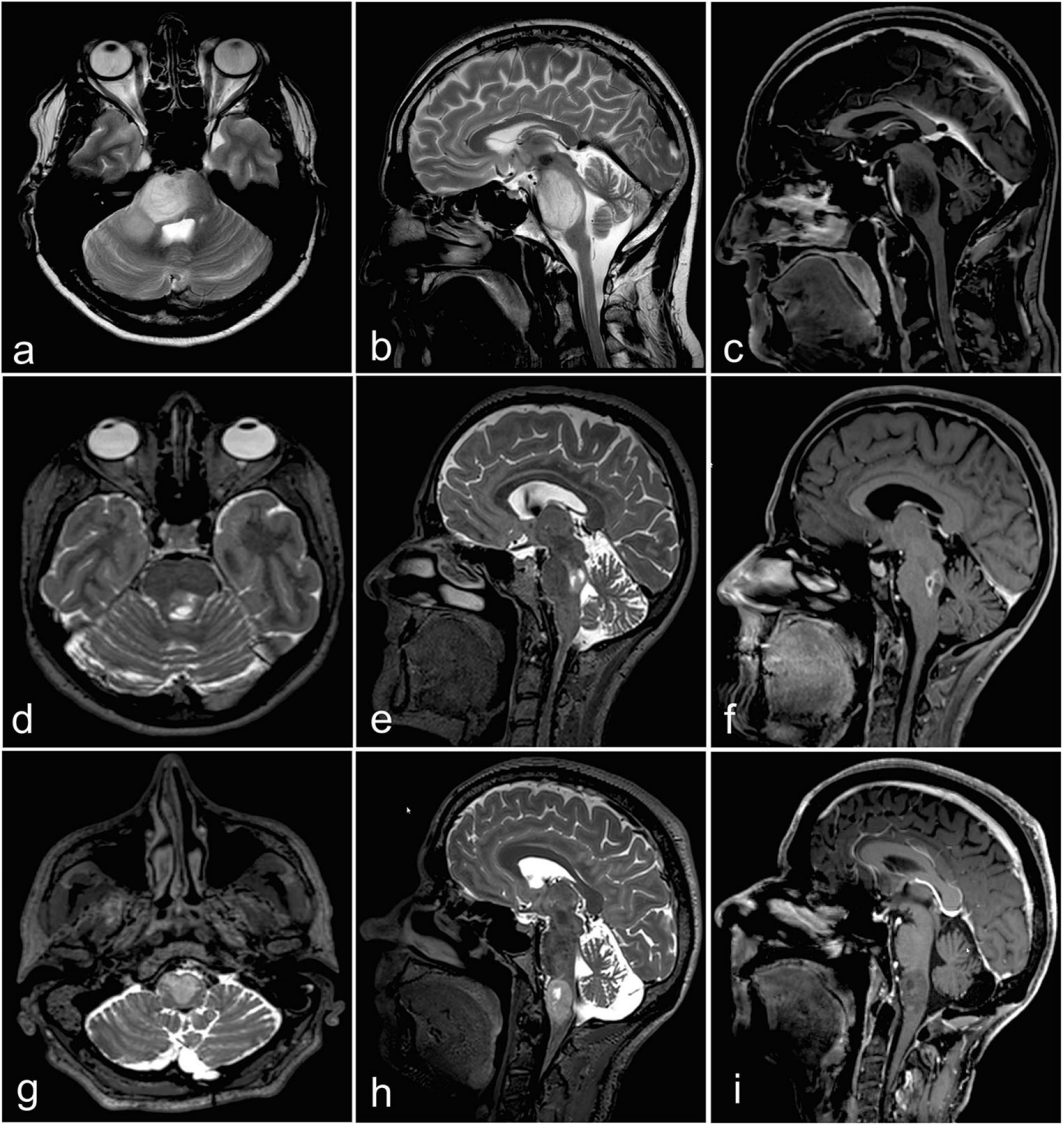
Overview of variety of some of the lesions that were biopsied. **a**–**c** T2W hyperintense, non-enhancing lesion of the pons. Histopathological diagnosis was high-grade glioma. **d**–**f** T2W hyperintense, enhancing lesion of the pons tegmentum. Histopathological diagnosis was astrocytoma WHO grade II. **g**–**i** T2W hyperintense mass, with enhancing foci of the medulla oblongata. Histopathological diagnosis was ependymoma WHO grade II

All patients underwent preoperative 3 Tesla MRI sequences including 3D T1-weighted MPRAGE with contrast and 3D T2-weighted sequences (voxel size 1 mm3) (Somaton, Siemens, Erlangen, Germany). Magnetic resonance spectroscopy (MRS) could be performed in only 11 patients due to uncooperativeness of the patients. FDG-PET scan has been performed in 5 cases where there was no clear enhancement and/or because of reimbursement issues. Figure 1 illustrates examples of brainstem lesions that were biopsied.

A stereotactic CT with contrast (voxel size 1 mm^3^) with the Leksell stereotactic frame (Elekta, Stockholm, Sweden) was performed on the day of surgery.

### Trajectory planning

**Fig. 2:**
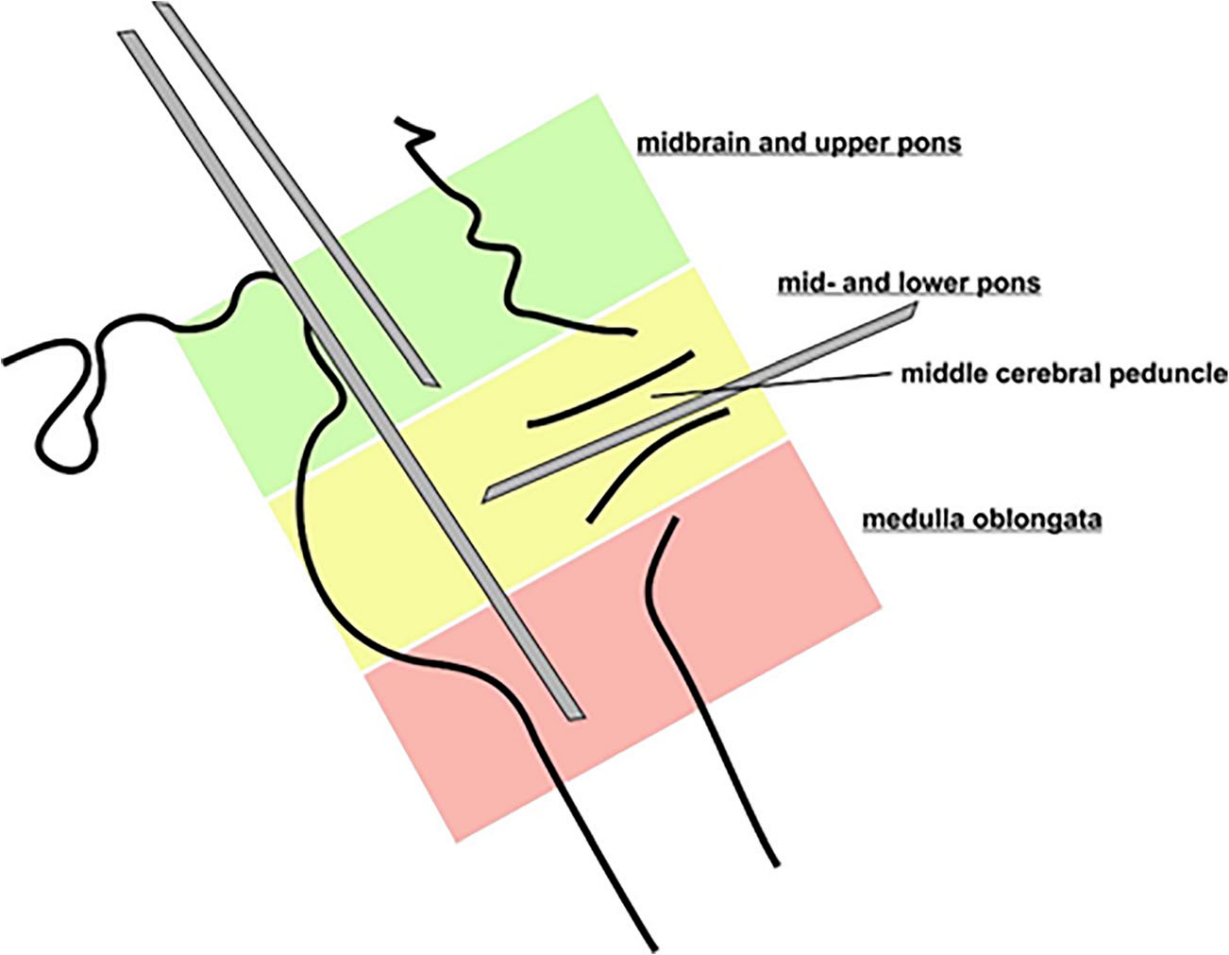
Diagram showing the selected approach according to the anatomical location of the lesion in the different brainstem regions

Surgical planning was performed with iPlan software (BrainLab, Germany). The target was chosen in the center of the contrast enhancing tumor or—in case of no enhancement—in the center of the brightest T2 signal abnormality of the lesion. We chose a supratentorial transfrontal trajectory in cases where the lesion was in the midbrain, upper pons, and medulla oblongata. Lesions in the lower pons that were accessible via the cerebellar peduncles were targeted via an infratentorial, transcerebellar, and transpeduncular approach (Fig. 2). All trajectories were planned to avoid blood vessels, sulci, and the ventricles.

### Surgical procedure

The surgical procedure was performed under general anesthesia. Minimal shaving is limited to the entry point area. DIXI guides tubes (DIXI, Besançon, France) were used to perform a 2.5-mm diameter burr hole strictly following the axis of the defined trajectory and then insert the stereotactic needle used for the biopsy.

In case of supratentorial transfrontal approach, the patient was placed in a standard supine position, whereas semi-sitting position was provided for transcerebellar approach. In some of our transcerebellar cases, we removed one of the rear bars of the frame in order to be able to perform the surgically planned approach. Usually, three samples were collected for each patient by rotating the opening of the biopsy needle into different direction and taking the subsequent samples but staying within the same initially chosen target point. The first specimen was used for frozen section processing to ensure positive sampling. The second or the third specimen was used for the definitive histological and molecular analysis or microbiology if an infectious process was suspected. The average time for the surgical procedure including the time needed for the frozen section assessment by the pathologist was 45 min.

Routine postoperative imaging was not applied but reserved for cases presenting a new neurological deficit after the procedure or confirming that the tissue sample was collected according to the planned target in case of diagnostic failure.

## Results

### Lesion location and trajectory

The location of the lesion according to epicenter of contrast enhancement or the T2-hyperintensity is shown in Table 2.

**Table 2:** Anatomical distribution of the lesions within the brainstem

Localization	Number (%)
Midbrain	9 (39)
Pons	2 (9)
Medulla	1 (4)
Diffuse brainstem	1 (4)
Pontomedullary	5 (22)
Pontomesencephalic	5 (22)

**Fig. 3:**
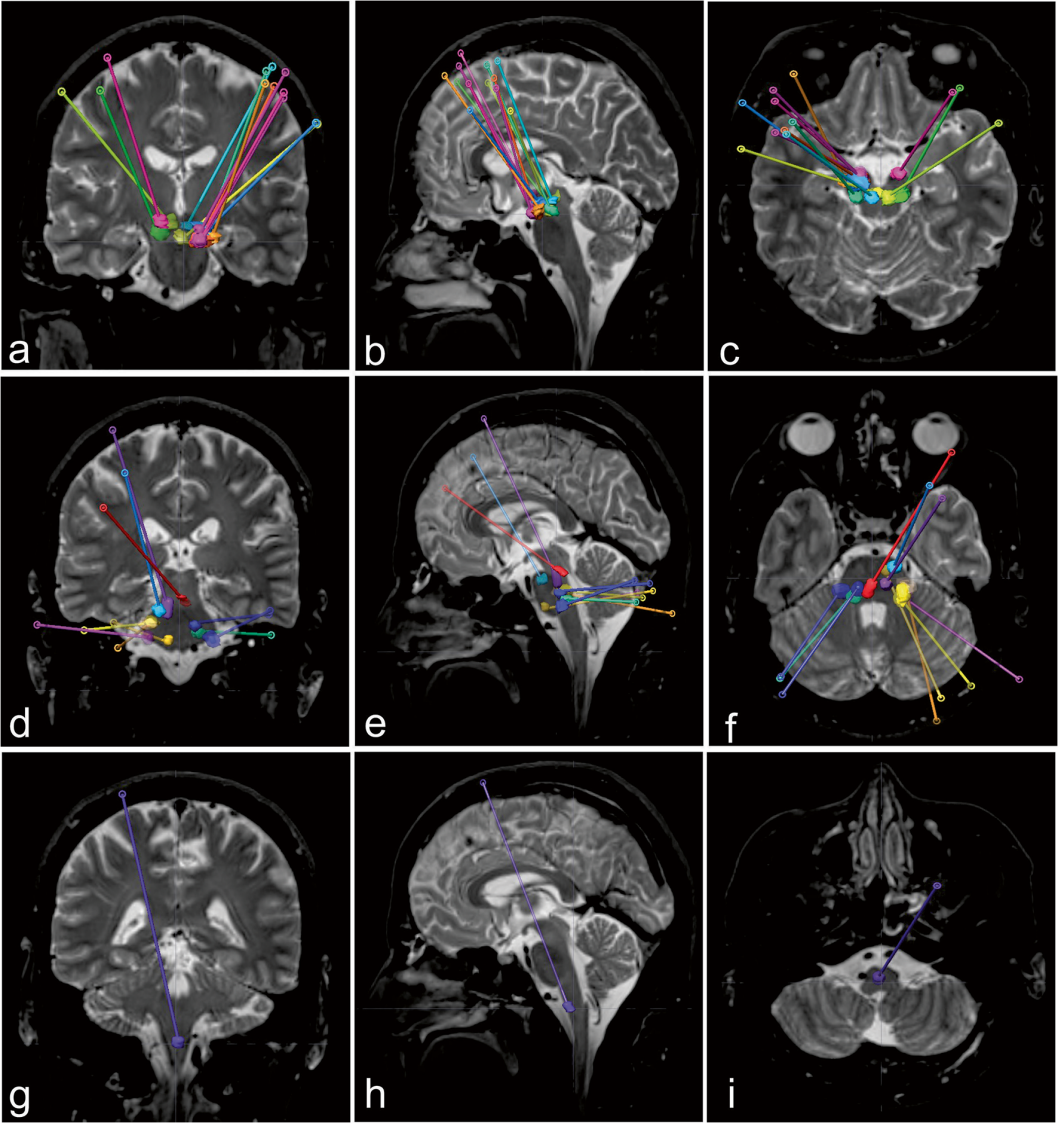
Schematic coronal (left), sagittal (middle), and axial (right) representation of the cases and the used trajectories, midbrain cases (**a**, **b**, **c**), pons cases (**d**, **e**, **f**), and medulla cases (**g**, **h**, **i**)

We applied a supratentorial transfrontal approach in all our cases of midbrain lesions, as the transcerebellar transpeduncular approach is limited because of the narrow superior cerebellar peduncle and the very low entry point beyond the limits of the frame ring angle (Fig. 3a, b, c). For pontine lesions, we used a supratentorial transfrontal approach in 30% of the cases and infratentorial transcerebellar transpeduncular approach in 70% (Fig. 3d, e, f). The supratentorial transfrontal approach was used in one case of medullary lesion as a transcerebellar transpeduncular approach was technically not feasible due to a resulting entry point above the tentorium (Fig. 3g, h, i).

Considering the whole cohort of patients, the transfrontal approach was used in 16 (70%) cases and the transcerebellar transpeduncular approach was used in 7 (30%) cases.

### Surgical outcome

There was no mortality in this series. Three patients developed transient neurological worsening which recovered spontaneously a few days later without any further neurosurgical intervention. One patient developed a contralateral upper and lower limb weakness after a biopsy of an astrocytoma WHO grade III located in the pontomesencephalic region. In this case, the biopsy was at cerebral peduncle taken via a supratentorial transfrontal approach. The other two patients developed an upper limb weakness after a biopsy of a lymphoma and a high-grade glioma (WHO grade IV). Both lesions were located in the midbrain tegmentum and the biopsy was taken via a supratentorial transfrontal approach. The immediate postoperative CT scan did not show any signs of hemorrhage, edema, or ischemia. No complications were found with the infratentorial, transcerebellar-transpeduncular approach.

### Histological diagnosis

In all patients, the initial frozen sample confirmed pathological tissue sampling. A conclusive definitive diagnosis was made in 21 patients (91%). Confirmed histological diagnoses are shown in Table 3.

**Table 3:** Histopathological diagnosis of the biopsied brainstem lesions

Histopathology results	Number of patients (%)
Astrocytoma WHO II	1 (4.3)
Astrocytoma WHO III	4 (17)
Glioblastoma multiforme WHO IV	8 (35)
Lymphoma	5 (22)
Metastasis	1 (4.3)
Ependymoma	1 (4.3)
Pilocytic astrocytoma	1 (4.3)
Uncertain diagnosis	2 (9)

Of the remaining two patients, one showed increased cellularity with Rosenthal fibers and the other non-specific gliosis with microhemorrhage. In both cases, we performed a postoperative CT scan which confirmed that the biopsy was performed in the targeted area. Both patients were followed up closely with repeated radiological and clinical assessment without changes of the clinical status in both.

## Discussion

In this study, we present a systematic anatomical and clinical analysis of different trajectories for brainstem biopsies and propose an illustrative scheme to approach these technically demanding lesions. In this series of 23 patients, both the supratentorial and infratentorial approaches yielded a definitive pathological diagnosis in over 90% of the cases. Furthermore, both techniques were not associated with long-term morbidity or mortality. Transient neurological deterioration occurred in 13% of our patients who underwent a biopsy of midbrain lesions via a supratentorial transfrontal approach.

We used both supratentorial (transfrontal or transparietal) and transcerebellar approaches. Both approaches have been described and used by several groups [4, 5, 11, 15, 16, 22]. However, information about the choice of the trajectory related to their precise location in the brainstem has not been specifically addressed in previous studies.

Depending on the exact location of the lesion in the brainstem, we recommend the following approaches:

1. Midbrain lesions can be approached through a supratentorial transfrontal approach. Choosing a gyrus near the coronal suture as an entry point is feasible for anteriorly located lesions (crus cerebri, as well as anterior tegmentum) and may need to be adjusted more anteriorly (up to 2 cm anterior to the coronal suture) for lesions located in the tectum (Fig. 4).

**Fig. 4:**
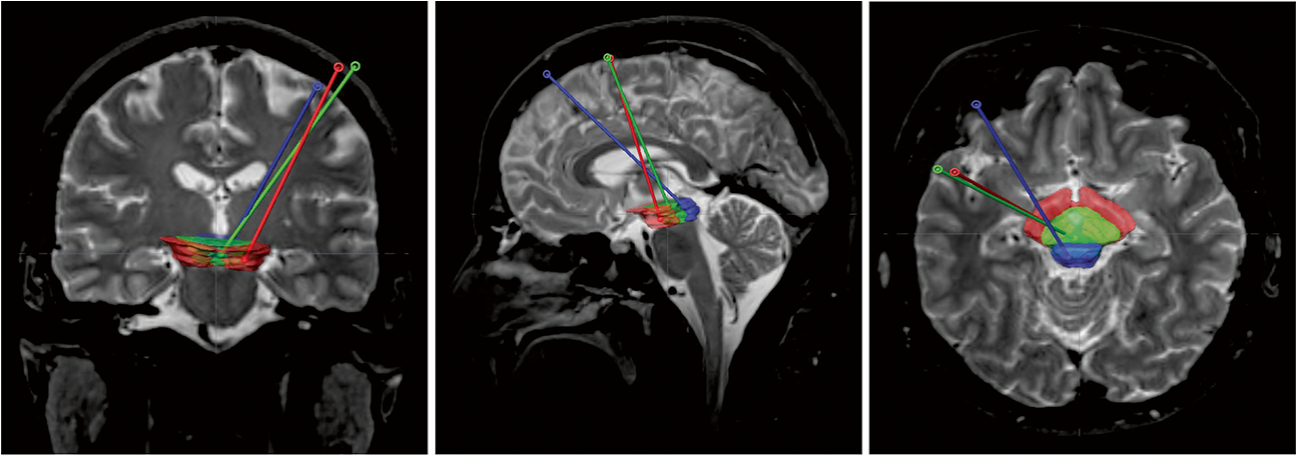
Coronal (left), sagittal (middle), and axial (right) T2-weighted MRI images showing the proposed midbrain regions of interest (ROI) and the preferred trajectory (red, crus cerebri; green, tegmentum; blue, tectum)
2. Lesions in the pons accessible through an infratentorial, transcerebellar-transpeduncular approach should preferentially be targeted by this approach due to the short trajectory and paucity of any eloquent tracts or nuclei along this trajectory. Superior pontine lesions as well as lesions in the midline of the pons are often not accessible through a transcerebellar-transpeduncular trajectory and a supratentorial transfrontal approach can be performed in these cases (Fig. 5).

**Fig. 5:**
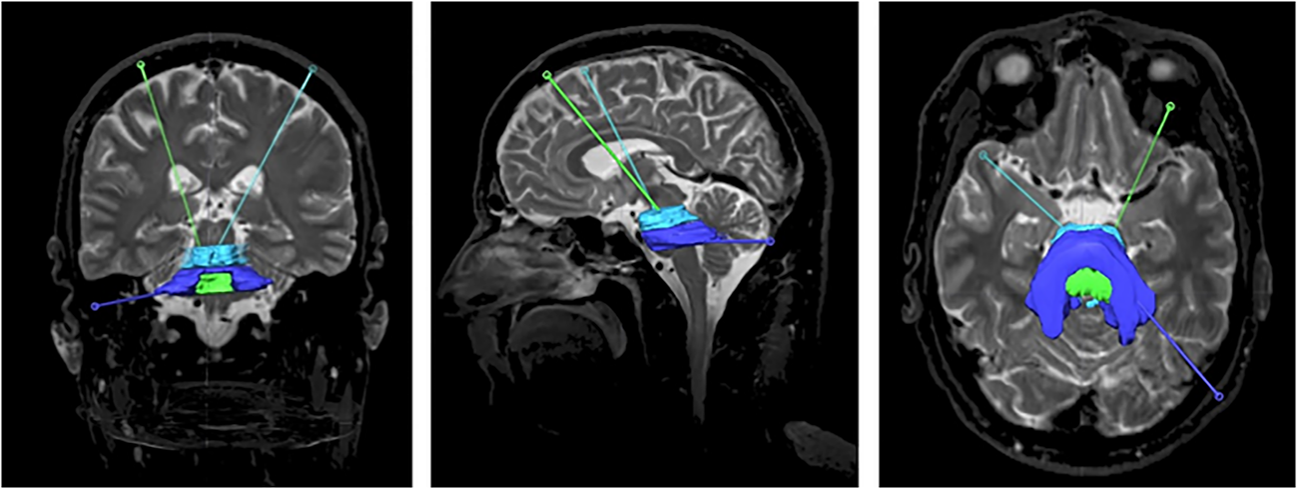
Posterior coronal (left), sagittal (middle), and axial (right) T2-weighted image showing the proposed pons regions of interest (ROI) and the preferred trajectory (light blue, upper pons; blue, pons at the level of the middle cerebellar peduncle; green, pons tegmentum)
3. Medullary lesions can be approached through a supratentorial transfrontal approach. Alternatively, in cases of a lesion located at the ponto-medullary junction, a transcerebellar-transpeduncular approach can be evaluated (Fig. 6). Selected superficial medullary lesions could also be biopsied through an open transcisternal approach.

**Fig. 6:**
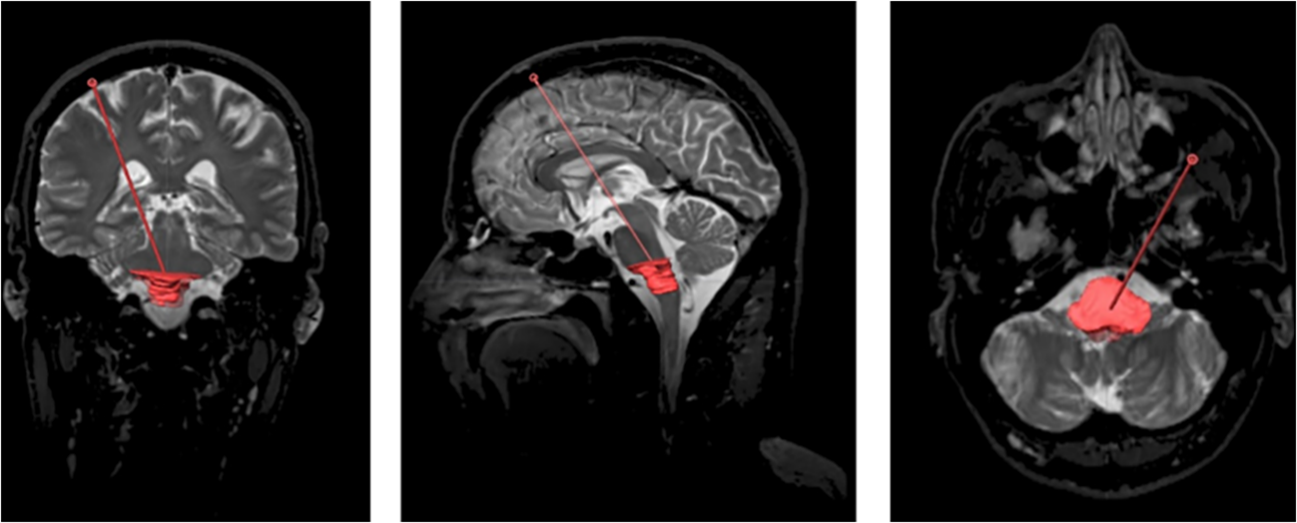
Coronal (left), sagittal (middle), and axial (right) T2-weighted MRI showing the medulla oblongata region of interest (ROI) and the proposed trajectory

We performed all our procedures under general anesthesia. Of note, most of the procedures described in literature were performed under local anesthesia and intermittent intravenous sedation agent. Based on our results, we think that performing brainstem biopsies under general anesthesia is safe and offers a more tolerable atmosphere for the patients. Furthermore, when performing procedures within the brainstem, respiratory compromise is unpredictable and sometimes catastrophic. Emergent intubation and resuscitation are occasionally required. We also strictly control the intraoperative blood pressure. It is impossible to compare our values with other data obtained from patients operated under local anesthesia, as these values are not reported. Therefore, we are not able to make any correlation about the intraoperative blood pressure value and the bleeding risk.

Some previous studies have reported on complication rates exceeding 10% [7, 10]. Furthermore, the diagnostic yield of stereotactic biopsy for intracranial lesions shows wide variations among different previous studies ranging from 2 to 30% in some studies [8, 14] and more than 90% in others. A recent meta-analysis of 38 studies reported a diagnostic yield of 96.2% by weighted average proportions analysis, which is comparable to our results [9]. Nevertheless, based on these observation, some authors have even questioned the use of stereotactic biopsy for these lesions [2, 3]. Interestingly, most of the lesions in the present study were located in the midbrain or upper pons and targeted with a supratentorial transfrontal approach and—consequently—a long trajectory tract. In contrast, in many previous studies, the majority of lesions were located in pons followed by medulla [7, 10, 20] but no details on the chosen trajectories are provided. We speculate that by using an infratentorial, transcerebellar-transpeduncular approach for mid-pontine lesion that come along with a shorter trajectory length within the brainstem should decrease the risk of bleeding or the risk of induced microlesion in that very eloquent area and could be accountable for the variability of observed complication rates.

Molecular markers of brain primary tumors such as O6-methylguanin-DNS-methyltransferase (MGMT), isocitratedehydrogenase-1 (IDH-1), and alpha-thalassemia/mental retardation syndrome X-linked (ATRX) have recently been shown to be correlated with chemotherapy response and prognosis [21]. These markers can only be determined by obtaining a tissue sample. This strongly argues for the important role of brainstem biopsies to not only confirm a suspected diagnosis but specify molecular subtypes of tumors with implications on subsequent therapeutic options.

There are some limitations for this study. The main limitation is the small sample size of patients of our cohort compromising a better estimate of complication rate and the diagnostic yield of brain stem biopsies in general. However, our results are in line with previous studies including larger numbers of patients [5, 11, 15, 16]. Furthermore, the current results are based only on observations reflecting our targeting strategy and no conclusions can be drawn if one of the two suggested approaches would yield lower complication rates. To answer this question, a prospective study or a retrospective case-control study of similar lesions targeted via different approaches and adequate sample size would be needed. On the other hand, we can demonstrate the feasibility of the two approaches with similar outcome. Although we would propose to apply a transcerebellar-transpeduncular approach whenever technically possible due to the shorter trajectory length and less eloquent brain tissue along the tract, of course, it remains the individual surgeon’s choice, which approach to apply.

## Conclusions

Stereotactic biopsies of brainstem lesions play an important role in the diagnosis and subsequent treatment of patients, especially in the current era of fine molecular diagnosis and targeted chemotherapy. Supratentorial transfrontal and transcerebellar-transpeduncular approaches can be applied and yield a high diagnostic accuracy and low morbidity. A specific trajectory according to the specific location of the lesion should be considered when approaching brainstem lesions. For midbrain lesion, we suggest a supratentorial transfrontal approach; for pontine lesion, a transcerebellar-transpeduncular trajectory should be the first choice if the lesion is reachable from that approach; and for medullary lesion, a transfrontal approach should be preferred unless the lesion is in the upper part of the medulla where transcerebellar transpeduncular approach can still be used.

## References

[1] Abernathey CD, Camacho A, Kelly PJ (1989). Stereotaxic suboccipital transcerebellar biopsy of pontine mass lesions. J Neurosurg.

[2] Albright AL (1996). Diffuse brainstem tumors: when is a biopsy necessary?. Pediatr Neurosurg.

[3] Albright AL, Guthkelch AN, Packer RJ, Price RA, Rourke LB (1986). Prognostic factors in pediatric brain-stem gliomas. J Neurosurg.

[4] Chen SY, Chen CH, Sun MH, Lee HT, Shen CC (2011). Stereotactic biopsy for brainstem lesion: comparison of approaches and reports of 10 cases. Journal of the Chinese Medical Association : JCMA.

[5] Dellaretti M, Reyns N, Touzet G, Dubois F, Gusmão S, Pereira JL, Blond S (2012). Stereotactic biopsy for brainstem tumors: comparison of transcerebellar with transfrontal approach. Stereotact Funct Neurosurg.

[6] Epstein F, McCleary EL (1986). Intrinsic brain-stem tumors of childhood: surgical indications. J Neurosurg.

[7] Guillamo JS, Monjour A, Taillandier L, Devaux B, Varlet P, Haie-Meder C, Defer GL, Maison P, Mazeron JJ, Cornu P, Delattre JY (2001). Brainstem gliomas in adults: prognostic factors and classification. Brain.

[8] Hall WA (1998). The safety and efficacy of stereotactic biopsy for intracranial lesions. Cancer.

[9] Kickingereder P, Willeit P, Simon T, Ruge MI (2013). Diagnostic value and safety of stereotactic biopsy for brainstem tumors: a systematic review and meta-analysis of 1480 cases. Neurosurgery.

[10] Kwon JW, Kim IO, Cheon JE, Kim WS, Moon SG, Kim TJ, Chi JG, Wang KC, Chung JK, Yeon KM (2006). Paediatric brain-stem gliomas: MRI, FDG-PET and histological grading correlation. Pediatr Radiol.

[11] Manoj N, Arivazhagan A, Bhat DI, Arvinda HR, Mahadevan A, Santosh V, Devi BI, Sampath S, Chandramouli BA (2014). Stereotactic biopsy of brainstem lesions: techniques, efficacy, safety, and disease variation between adults and children: A single institutional series and review. J Neurosci Rural Pract.

[12] Massager N, David P, Goldman S, Pirotte B, Wikler D, Salmon I, Nagy N, Brotchi J, Levivier M (2000). Combined magnetic resonance imaging- and positron emission tomography-guided stereotactic biopsy in brainstem mass lesions: diagnostic yield in a series of 30 patients. J Neurosurg.

[13] Pereira EA, Jegan T, Green AL, Aziz TZ (2008). Awake stereotactic brainstem biopsy via a contralateral, transfrontal, transventricular approach. Br J Neurosurg.

[14] Perez-Gomez JL, Rodriguez-Alvarez CA, Marhx-Bracho A, Rueda-Franco F (2010). Stereotactic biopsy for brainstem tumors in pediatric patients. Childs Nerv Syst.

[15] Puget S, Beccaria K, Blauwblomme T, Roujeau T, James S, Grill J, Zerah M, Varlet P, Sainte-Rose C (2015). Biopsy in a series of 130 pediatric diffuse intrinsic Pontine gliomas. Childs Nerv Syst..

[16] Quick-Weller J, Lescher S, Bruder M, Dinc N, Behmanesh B, Seifert V, Weise L, Marquardt G (2016). Stereotactic biopsy of brainstem lesions: 21 years experiences of a single center. J Neurooncol.

[17] Rachinger W, Grau S, Holtmannspotter M, Herms J, Tonn JC, Kreth FW (2009). Serial stereotactic biopsy of brainstem lesions in adults improves diagnostic accuracy compared with MRI only. J Neurol Neurosurg Psychiatry.

[18] Reyes-Botero G, Mokhtari K, Martin-Duverneuil N, Delattre JY, Laigle-Donadey F (2012). Adult brainstem gliomas. Oncologist.

[19] Samadani U, Stein S, Moonis G, Sonnad SS, Bonura P, Judy KD (2006). Stereotactic biopsy of brain stem masses: decision analysis and literature review. Surg Neurol.

[20] Selvapandian S, Rajshekhar V, Chandy MJ (1999) Brainstem glioma: comparative study of clinico-radiological presentation, pathology and outcome in children and adults. Acta Neurochir (Wien) 141(7):721–726 discussion 726–727 10.1007/s007010050367 10481783

[21] Weise LM, Harter PN, Eibach S, Braczynski AK, Dunst M, Rieger J, Bahr O, Hattingen E, Steinbach JP, Plate KH, Seifert V, Mittelbronn M (2014). Confounding factors in diagnostics of MGMT promoter methylation status in glioblastomas in stereotactic biopsies. Stereotact Funct Neurosurg.

[22] Zrinzo LU Thomas DGT (2009). Stereotactic approaches to the brain stem. Textbook of Stereotactic and Functional Neurosurgery. A. M. Lozano, P. L. Gildenberg and R. R. Tasker. Berlin, Heidelberg, Springer Berlin Heidelberg: 789–795.

